# NUTRItion and CLIMate (NUTRICLIM): investigating the relationship between climate variables and childhood malnutrition through agriculture, an exploratory study in Burkina Faso

**DOI:** 10.1186/s40985-016-0031-6

**Published:** 2016-10-06

**Authors:** Raissa Sorgho, Jonas Franke, Seraphin Simboro, Revati Phalkey, Rainer Saeurborn

**Affiliations:** 1 Institute of Public Health, Universitats Klinikum, Heidelberg, Germany; 2 Remote Sensing Solution GmBh (RSS), Environmental Consulting, Baierbrunn, Germany; 3 Centre de Recherche en Santé de Nouna (CRSN), Research Center, INDEPTH Network, HDSS, Nouna, Burkina Faso; 4 Division of Epidemiology and Public Health, University of Nottingham, Nottingham, UK

**Keywords:** Malnutrition, Low- and middle-income countries, Climate change, Stunting, Remote sensing, Weather

## Abstract

Malnutrition remains a leading cause of death in children in low- and middle-income countries; this will be aggravated by climate change. Annually, 6.9 million deaths of children under 5 were attributable directly or indirectly to malnutrition. Although these figures have recently decreased, evidence shows that a world with a medium climate (local warming up to 3–4 °C) will create an additional 25.2 million malnourished children. This proof of concept study explores the relationships between childhood malnutrition (more specifically stunting), regional agricultural yields, and climate variables through the use of remote sensing (RS) satellite imaging along with algorithms to predict the effect of climate variability on agricultural yields and on malnutrition of children under 5. The success of this proof of purpose study, NUTRItion and CLIMate (NUTRICLIM), should encourage researchers to apply both concept and tools to study of the link between weather variability, crop yield, and malnutrition on a larger scale. It would also allow for linking such micro-level data to climate models and address the challenge of projecting the additional impact of childhood malnutrition from climate change to various policy relevant time horizons.

## Main text

Malnutrition is globally recognized as having one of the largest adverse effects on the growth of nations, because it not only poses a challenge to the health but also to the productivity of populations [1]. Unfortunately, climate change will have an additional negative impact on childhood nutrition through a large number of factors [1, 2]. While malnutrition in children has globally decreased over the past few decades, climate change has the potential to reverse the recent gains in the global reduction of malnutrition [3]. A median climate (local warming up to 3–4 °C) is projected to create an additional 25.2 million malnourished children [2, 4]. The 2015 Rockefeller Foundation and Lancet Commission on Planetary Health publication titled: Safeguarding human health in the Anthropocene epoch, states along with the IPCC that “… median crop yields would decrease by 0–2 % per decade for the remainder of the century, as a result of climate change alone, with or without adaptation, whereas demands for crops are projected to increase by 14 % per decade up to 2050” [5, 6]. The publication continues by detailing that the projected decreases in crop yields result in increasing numbers of stunted children, especially in Asia and Africa. More than 90 % of the world’s stunted children live in Africa and Asia [7]. Currently, 36 % of all children under 5 years in sub-Saharan Africa suffer from stunting—a severe form of malnutrition [1, 2]. Projections forecast that stunting will increase by approximately 23 % in the region; this is why we selected a sub-Saharan African country as the site of this proof of concept exploratory study [2].

**Fig. 1:**
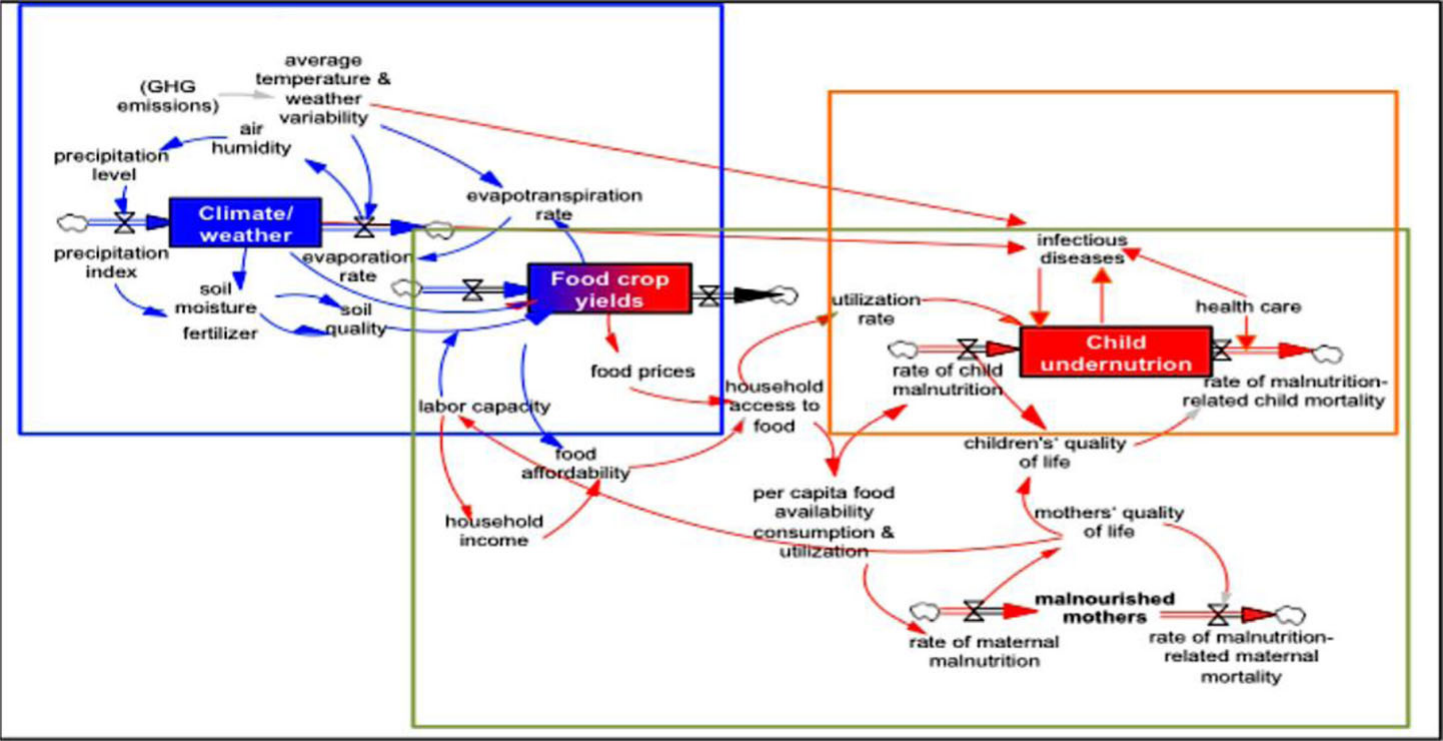
The complexity and interdisciplinary nature of these three issues converge into an elaborate web. Adapted from Phalkey et al. [1]

The relationship between changing climate, agriculture, and malnutrition is influenced by a multitude of factors. The complexity and interdisciplinary nature of these three issues converge into an elaborate web, which is represented in Fig. 1. The convolution of these links is precisely the reason these connections have been understudied.

Figure 1 illustrates an aspect of the complexity of the relationships investigated. Each of the three colored boxes represents one of three pillars: climate, agriculture, and malnutrition. Each arrow represents a relationship between two variables or factors. The three pillars converge on the subject of food crops and food yields.

This exploratory study, NUTRItion and CLIMate (NUTRICLIM), in Burkina Faso aims to investigate the relationship between weather variability, crop yields, household socioeconomic variables, and malnutrition. The study village of Bourasso, which has 12,548 inhabitants, in the rural Kossi Province is located 25 km from the small town of Nouna. The study involves 156 individuals, subdivided into 20 households with 29 children under the age of 5. This first sample was randomly selected from the INDEPTH Human Demographic Surveillance System (HDSS) database of the Centre de Recherché en Santé de Nouna (CRSN) and the second sample from local Bourasso Health Post’s database of malnourished children [8]. The 20 households can thus be subdivided into sample 1: 10 households that were randomly selected and coincidentally had no malnourished children under the age of 5, and sample 2: 10 households that were purposefully selected for having children under the age of 5 who were undergoing treatment for malnutrition. The additional selection criteria were that all 20 households be subsistence farmers, living in the village of Bourasso, with at least one child under the age of 5.

Data for the first pillar, climate, were acquired through the two nearest local weather stations of the HDSS. The weather stations provide information on median daily temperature, daily rainfall, as well as seasonal distribution and variability of rainfall.

Data for the second pillar, agriculture, was collected through two methods. The first method was harvest yields reported by farmers and converted from local measures into kilograms. The second was innovative in that it estimated plot level yields by household and crop using special algorithms from remotely sensed data of the village and its surroundings. This required the delineation of each field of all households with the farmers using a GPS to establish the polygons. These were overlaid with scenes from the RapidEye satellite, covering the agricultural fields of the 20 selected households. We carried out ground validation through verification and comparison of the results from remote sensing (RS) satellite imaging; field agents physically verified that the satellite readings matched ground data. Post-harvest, the figures of the actual agricultural yields are used as input data to better calibrate the algorithms for modeling crop yields on a micro-level (household level).

The third pillar covered malnutrition and health. This data was collected using a socioeconomic and morbidity questionnaire for the selected households. The survey assessed (i) household assets, revenues, and expenditure, (ii) a 24-hour nutritional recall journal of all children under 5, and (iii) all recent child illnesses within the household, both chronic and acute (episodes of diarrhea, malaria, etc.). Furthermore, we used standard anthropometry (weight, height, and mid-upper-arm circumference) to assess the nutritional status of children under the age of 5.

As data analysis was still in progress at the time of the presentation at the COP21, no definitive results could be stated. But preliminary findings indicate the possibility of disparities in the agricultural yield of households with and without malnourished children and between years with average and low rainfall. The differences were noticed not only in the types of crops sown but also in the number of plots owned by the households: households with healthy children had, on average, a greater number of fields. When the subsistence farmers were questioned on their yields, only one third classified their harvest as good, allowing the household to be fed in a satisfactory manner for the entire year until the next harvest. The remaining two thirds of all households attributed insufficient yields to bad rains, changing rain patterns, or unpredictable rain patterns. This potentially highlights the significance of changing weather patterns and their consequences in terms of droughts [4, 9].

## Conclusions

We laid out a number of field methods in the fields of meteorology, agriculture, nutrition, and health that allow for the study of the web of causation of childhood malnutrition with a particular focus on the role of weather and climate in the future.

We propose that large-scale studies using these methods, amongst others, be considered. These could then be linked to downscaled climate models in cooperation with climate scientists in order to establish data-based projections of the future impact of climate change on malnutrition rather than relying on a set of assumptions and mono-disciplinary fragmented studies.

## Abbreviations

CRSNm: Centre de Recherche en Sante de Nouna
HDSS: Human Demographic Surveillance System
